# Performance of GPT-based large language models in hepatocellular carcinoma stratification: liver function assessment, BCLC staging, and treatment recommendations

**DOI:** 10.1038/s41598-026-56992-7

**Published:** 2026-06-12

**Authors:** Max Masthoff, Amelie Zipser, Michael Praktiknjo, Jonel Trebicka, Haluk Morgül, Andreas Pascher, Gesa Pöhler, Michael Köhler, Philipp Schindler

**Affiliations:** 1 Clinic for Radiology, University and University Hospital of Münster, Münster, Germany; 2 Department of Internal Medicine B, University of Münster, Münster, Germany; 3 Department of General, Visceral and Transplant Surgery, University of Münster, Münster, Germany

**Keywords:** HCC, BCLC staging, Large language model, GPT, Artificial intelligence, Cancer, Gastroenterology, Oncology

## Abstract

Large language models (LLMs) like GPT have been proposed to support complex clinical decision-making. This study evaluated the performance of GPT-based LLM in analyzing clinical, radiological, and laboratory data from patients with hepatocellular carcinoma (HCC) to assess liver function, assign BCLC stage, and recommend treatment. Data from 106 HCC patients (82% male, median age 65 [22–86]) were compiled into anonymized integrated reports. Four GPT-versions (4, o1, o3, 5.4) were prompted—using both short and long instructions—to calculate MELD, ALBI, and Child–Pugh scores, assign BCLC stage, and generate treatment recommendations based on current guidelines. Outputs were compared to expert consensus and tumor board decisions. Errors were categorized by type and source. Time and cost analyses compared GPT to clinical staff. All GPT versions achieved high accuracy (> 85%) in liver function assessment, with MELD calculation being the most error-prone. BCLC staging accuracy ranged from 46.2% (version 4) to 84.0% (o3), with misclassification of radiological reports as the main error source. Reasoning-optimized models (o1, o3) performed best for treatment recommendations, achieving an overall accuracy (correct suggestions and acceptable alternatives) of up to 90.6%. In 9–14% of cases, GPT suggestions were retrospectively more guideline-concordant than tumor board decisions. GPT processing was significantly faster and reduced costs by approximately 300- to 1300-fold compared to clinical staff. GPT-based LLMs show potential as decision-support tools for liver function assessment, BCLC staging, and treatment guidance in HCC. Particularly with reasoning-optimized models and detailed prompting, LLMs may serve as valuable adjuncts in multidisciplinary HCC workflows. However, a non-negligible error rate requires expert oversight and further model refinement.

**Supplementary Information:**

The online version contains supplementary material available at 10.1038/s41598-026-56992-7.

## Introduction

Hepatocellular carcinoma (HCC) is the most common primary malignancy of the liver and one of the leading causes of cancer-related mortality worldwide^1^. Its management demands a multifaceted approach, integrating tumor burden, liver function, and patient performance status. The Barcelona Clinic Liver Cancer (BCLC) staging system remains the cornerstone of HCC stratification, offering a framework for both prognosis and treatment planning^2^. Whereas tumor burden assessment relies primarily on radiological data—encompassing lesion number and size, local infiltration, and metastatic spread—the determination of preserved versus non-preserved liver function depends on established clinical and laboratory scores, including the Albumin-Bilirubin (ALBI) grade^3^, Child–Pugh score^4,5^, and Model for End-Stage Liver Disease (MELD) score^6^. Consequently, accurate staging and optimal treatment decisions require a comprehensive integration of clinical, laboratory, and radiological information to select the most appropriate individualized treatment strategy. However, these data are frequently communicated in disparate formats: clinical and radiological findings in prose-text documents, and laboratory data in parameter-based tables. Such heterogeneity does not automatically consolidate the required information to derive liver function scores, assign BCLC stages, or determine treatment pathways. As a result, each discipline must manually compile and interpret these data before interdisciplinary tumor board discussions, a process that is both time-intensive and prone to errors or incomplete information. Thus, decision-support tools integrating these data would highly desirable in HCC management.

Recent advancements in artificial intelligence and natural language processing have spurred the exploration of large language models (LLM), such as GPT, for medical applications^7–10^. GPT-is a LLM integrated into an artificial intelligence chatbot developed by OpenAI (San Francisco, California, US), and has demonstrated capabilities in generating coherent medical information, summarizing literature, improve workflow efficiency, assist in clinical decision-making and providing clinical insights and facilitate education across diverse specialties^11–17^. While GPThas been evaluated for various oncology applications^17^, its role in the nuanced stratification of HCC patients remains underexplored. The ability to integrate clinical data such as ALBI grade, Child–Pugh or MELD score to assess liver function, patient performance status, and tumor characteristics, and then align these metrics with BCLC staging criteria and corresponding treatment recommendations, could represent a major advancement by improving workflow efficiency, reducing errors, and enhancing the consistency and timeliness of therapeutic decisions.

This study aims to evaluate GPT’s potential for comprehensive HCC patient stratification according to the BCLC staging system by integrating liver function, tumor burden, and performance status data. Additionally, it investigates the model’s ability to provide treatment recommendations aligned with current clinical guidelines. By examining GPT’s accuracy and reliability in these tasks, this research explores its viability as a decision-support tool in the complex management of HCC.

## Methods

### Study design

This retrospective single-center study included all consecutive patients with HCC who were newly referred to a tertiary care liver center and underwent comprehensive clinical, radiological, and laboratory evaluation between January 2021 and October 2024. HCC diagnosis was made in all patients according to the European Association for the Study of the Liver (EASL) guidelines^18^. Our center offers the full range of possible treatment options for HCC, including liver transplantation, resection, local percutaneous strategies (radiofrequency and/or microwave ablation), local endovascular strategies (transarterial chemoembolization, TACE and/or transarterial radioembolization, TARE), systemic therapy or best supportive care. Patients were excluded from the study if they had undergone HCC therapy at another center within the last 3 months or if their clinical, radiological, or laboratory data were incomplete, as specified below.

The study was conducted in accordance with the Declaration of Helsinki. For this retrospective study, the requirement for written informed consent was formally waived, and the study was approved by the Ethics Committee Westfalen-Lippe of the University of Muenster (ID: 2024–546-f-N).

### Data collection and management

All relevant clinical, laboratory and radiological data were systematically collected, with specific mandatory minimum requirements for each report. Clinical data reports had to include information on co-morbidities, a history and grading of hepatic encephalopathy according to the West-Haven Classification^19^, and the tumor-related ECOG performance status. Laboratory data reports were required to contain at least albumin, bilirubin, creatinine, alpha fetoprotein (AFP), thrombocytes, prothrombin time and the International Normalized Ratio (INR). Radiological reports had to provide a minimum of essential details, including the number, size, shape (nodular or diffuse) and location of all HCC lesions, the presence or absence of macrovascular invasion, ascites, and lymphatic or hematogenous metastases. Additionally, radiological imaging protocols were reviewed for adherence to national HCC diagnostic guidelines. Compliance was assessed based on appropriate acquisition of contrast phases (arterial, portal venous, and late venous) in liver imaging (CT or MRI) and staging examinations of the chest (CT) and abdomen (CT or MRI). The Picture Archiving and Communication System (PACS, Centricity Universal Viewer, GE Healthcare Technologies, Chicago, USA) was used for this evaluation. Patients with any missing clinical, laboratory, or radiological data were excluded from further analysis. For those included, the complete original clinical, radiological, and laboratory reports were retrieved as PDF files from the Clinical Information System (CIS; Orbis, Dedalus, Milan, Italy) and compiled into a single anonymized PDF file in German language, with all patient- and physician-identifying information removed. No structured imaging metadata was included in the integrated reports. We recorded the time taken to collect clinical, radiological and laboratory results and to check available data for the mandatory information as defined above.

The BCLC classification was not included in the final integrated reports but was documented separately, as assigned according to the current BCLC guidelines^2^ and performed by an interdisciplinary expert panel (radiology, hepatology, surgery with each member having at least 5 years of experience in HCC management) in consensus, blinded for GPT outputs and provided with the same integrated report used for GPT analysis. Consensus data served as the gold standard for subsequent data analysis by GPT.

A crucial part of the BCLC classification system is the judgment on preserved or non-preserved liver function, reflected by a sum of scoring systems such as MELD, ALBI grade or Child–Pugh score^3–6^. These scores, all based on clinical, radiological and especially laboratory data available in the integrated reports, were not included in the reports. We calculated the MELD and ALBI scores using established calculators according to the following formulas^20,21^: MELD = (0.957 × ln(Serum creatinine) + 0.378 × ln(Serum bilirubin) + 1.120 × ln(INR) + 0.643) × 10 and ALBI = (log10 bilirubin × 0.66) + (albumin ×  − 0.085). Finally, the Child–Pugh class was calculated as established previously, with a score of 5–6 classified as stage A, 7–9 as stage B and 10–15 as stage C^4,5,22^. The retrieved MELD, ALBI grade and Child–Pugh score were available to the interdisciplinary expert panel for BCLC classification and served as gold standard for subsequent analysis by GPT.

Finally, the treatment decisions made by the interdisciplinary tumor board were also extracted from the CIS for all included patients. Treatment decisions were classified as (1) liver transplant evaluation with or without bridging therapy such as local ablation or endovascular treatment; (2) liver transplant evaluation with downstaging therapy using local ablation or endovascular treatment; (3) surgical resection; (4) endovascular therapy, including TACE, TARE, or radiation; (5) systemic therapy; and (6) best supportive care.

### GPT data analysis

The anonymized, integrated reports containing clinical, laboratory, and radiological data for each patient were analyzed using the LLM GPT (OpenAI, San Francisco, USA) using versions 4, o1 and o3, performed in December 2024 as a well as version 5.4 (thinking mode; thinking effort: standard) performed in April 2026. Using "zero-shot" conditions, the LLMs were not pre-trained or optimized for a particular domain^23^. No preprocessing scripts were used. The analysis involved three primary tasks: (1) calculating established clinical scores for liver function assessment, including the MELD score, ALBI grade, and Child–Pugh score; (2) classifying the patient according to the latest BCLC classification system; and (3) suggesting an appropriate treatment regimen.

To evaluate whether a concise, imperative task description was sufficient for the LLM to accurately analyze the data or if a more detailed prompt was necessary, GPT was tested using either a short prompt (SP) or a long prompt (LP) for each task. The prompts were applied as follows:

#### Short prompt (SP)

“*Using the attached patient information for a diagnosis of hepatocellular carcinoma (HCC), determine the ALBI grade, Child-Pugh score, MELD score and BCLC stage. Based on the newest BCLC system, recommend an appropriate treatment regimen.*”

#### Long prompt (LP)

“*Using the attached patient data for a diagnosis of hepatocellular carcinoma (HCC), perform the following tasks:*

1. *Determine Scores and Grades:*
  *Calculate the ALBI score using the formula:*
    *ALBI = (log10 bilirubin [μmol/L] × 0.66) + (albumin [g/L] × −0.085). Classify the ALBI grade as:*
    Grade 1: ALBI Score ≤  − 2.60
    Grade 2: ALBI Score >  − 2.60 to ≤  − 1.39
    Grade 3: ALBI Score >  − 1.39.
  Determine the Child–Pugh score and MELD score using appropriate scoring systems. Calculate the MELD Score using the formula:
    *MELD = 10 × (0.957 × ln(creatinine [mg/dl]) + 0.378 × ln(bilirubin [mg/dl]) + 1.12 × ln(INR) + 0.643)*
    *Consider if any score is <1, the MELD assumes the score is equal to 1.*
  Ensure all calculations and classifications adhere to the correct units and laboratory values.
2. *Classify Disease Stage:*
  *Assign the patient to the correct BCLC stage by conducting a comprehensive assessment of tumor burden, liver function, and performance status (considering only in case of tumor-related symptoms). Follow the 2022 BCLC guidelines strictly, incorporating key updates such as classifying single tumors as BCLC A regardless of size, provided liver function and performance status are adequate.*”
3. *Recommend Treatment:*
  *Provide a treatment recommendation based on the latest BCLC guidelines, considering the full spectrum of options, including combination therapies, bridging strategies, downstaging concepts, and stage migration approaches.*”

All calculations and results generated by the LLMs were documented using Microsoft Excel (Microsoft, Redmond, USA). Finally, an error analysis of GPT for each task was performed. Errors were defined as any deviation from the gold standard either by wrong or ambiguous GPT output. In the error analysis of liver function assessment (via Child–Pugh, MELD, and ALBI), four types of errors were considered: (1) source/formula-based errors, (2) text/data interpretation errors, (3) calculation errors, and (4) a combination of at least two of these categories. For BCLC classification, errors were categorized as, (1) errors in liver function assessment (i.e., carryover from the previous task), (2) errors in classification of radiological data (i.e. determining lesion number or size, metastases etc.), (3) errors in clinical data assessment (e.g., tumor-related ECOG performance status), or (4) a combination of at least two of these categories.

Treatment suggestions by GPT were categorized into the above mentioned six groups and were rated either true, false or as acceptable alternative based on comparison to the gold standard and BCLC classification (see above). GPT outputs were further retrospectively compared to real-world multidisciplinary board decision.

### Time efficiency and cost analysis

All steps were time-tracked using a digital stopwatch. For GPT, measurement began when a task was submitted and ended once the model completed its response. In the case of the multidisciplinary board (gold standard), each task was timed individually, and the total duration was calculated.

To enable an economic comparison between GPT and human experts, wage data from German university hospital collective labor agreements were used. Specifically, hourly rates were calculated for a resident physician with 3 years of experience ($0.64/min) and an attending physician with 4 years of experience ($1.03/min) using the exchange rate from February 26, 2025 (1 USD = €0.95). At the time of the study, the cost of a GPT team license was $25 per user per month. This corresponds to approximately $0.003 per minute of usage (assuming comparable working hours to human staff to reflect manual GPT operation). To calculate the cost per case for each scenario, the mean time in minutes spend was multiplied with the per minute rates.

### Statistical analysis

Data are presented as total number and percentage or mean and standard deviation (SD), as appropriate. Graphical illustration was performed using GraphPad Prism (Dotmatics, Boston, Massachusetts, USA) and biorender.com (Toronto, Ontario, Canada). Statistical analysis was performed using SPSS Statistics version 29 (SPSS Inc., Chicago, IL, USA). First, a Chi-square test was performed to assess whether the proportion of correct vs. incorrect calculations differed across multiple GPT versions. Upon finding a global significant difference (*p* < 0.05), pairwise column proportion z-tests were conducted within the crosstabs procedure, applying a Bonferroni correction to control for multiple comparisons. In the resulting output, versions that did not share a letter subscript were deemed significantly different at the 5% significance level.

## Results

### Study cohort

This study included anonymized integrated reports from 106 patients with HCC, comprising 87 men (82%) and 19 women (18%), with a median age of 65 years (range, 22–86). Among these patients, 65 (61%) were classified as Child–Pugh class A, 34 (32%) as class B, and 7 (7%) as class C. In terms of ALBI grade, 34 (32%) were grade 1, 60 (57%) grade 2, and 12 (11%) grade 3, while the mean (± SD) MELD score was 12.2 ± 5.9. With respect to BCLC stage, 4 patients (4%) were classified as stage 0, 43 (41%) as stage A, 24 (23%) as stage B, 22 (21%) as stage C, and 13 (12%) as stage D. Detailed patient characteristics are shown in Table 1.

**Table 1:** Patient characteristics.

Study cohort	
Total (%)	106 (100)
Sex	
Male	87 (82)
Female	19 (18)
Age	64.5 (22–86)
Initial treatment status	
Initial diagnosis	96 (91)
Relapse	1 (1)
Prior treatment > 3 months ago	9 (8)
Child–Pugh-score	
A	65 (61)
B	34 (32)
C	7 (7)
ALBI-grade	
1	34 (32)
2	60 (57)
3	12 (11)
MELD-score	12.2 (5.9)
BCLC	
0	4 (4)
A	43 (41)
B	24 (23)
C	22 (21)
D	13 (12)

Values denote n (%), mean (SD), median (range).

### GPT for liver function analysis

We challenged GPT to calculate the Child–Pugh Score, MELD score, and ALBI grade from the information provided in the integrated reports.

**Fig. 1:**
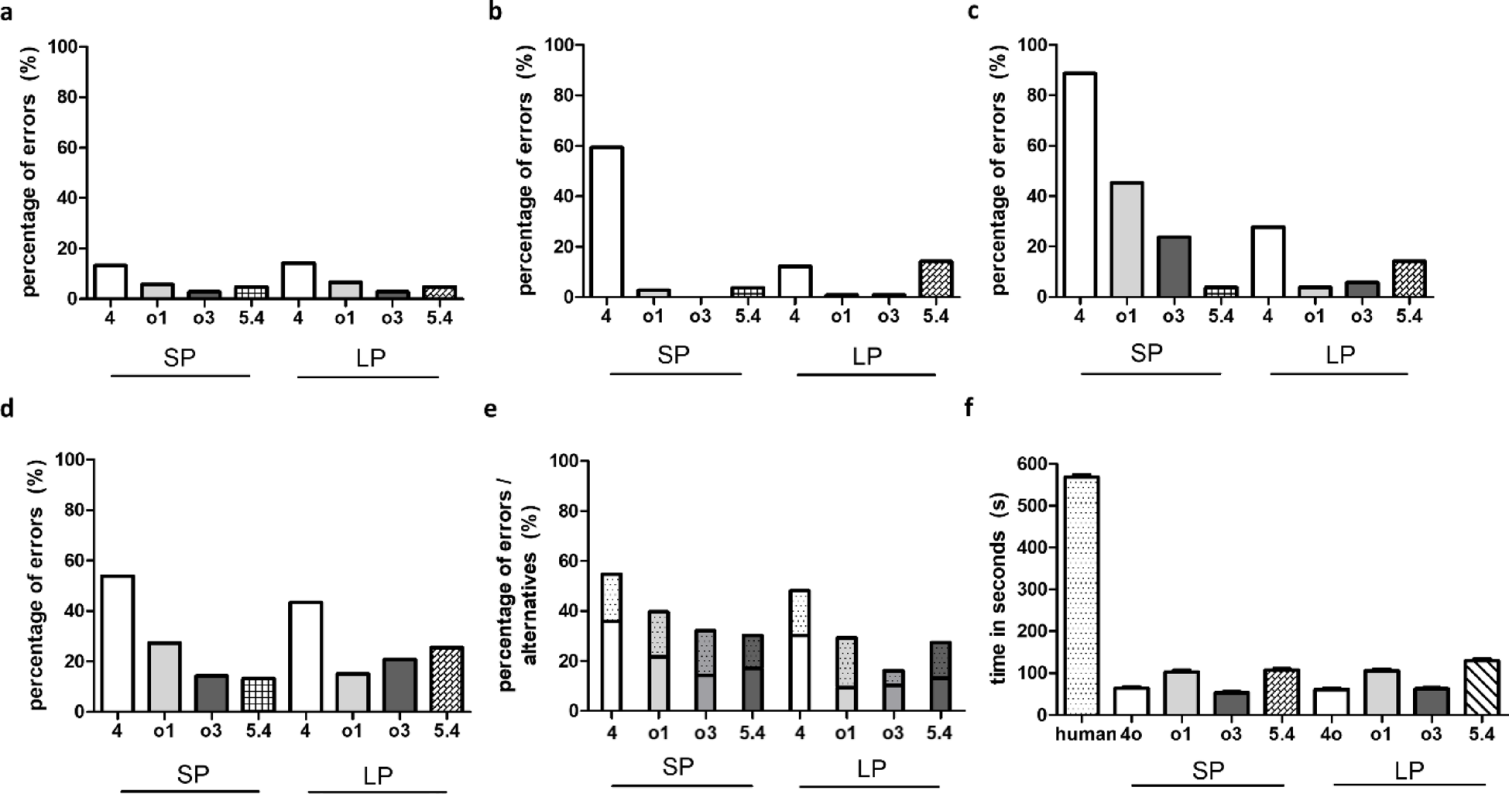
Performance of GPT for liver function assessment, BCLC staging and treatment guidance. (**a**–**e**) Error rate in (%) of different GPT versions (4, o1, o3, 5.4) with a short (SP) or long prompt (LP) for calculating, (**a**) Child–Pugh score, (**b**) ALBI grade, (**c**) MELD score, or performing, (**d**) BCLC staging or (**e**) treatment recommendations. Dotted area in (**e**) reflects rate of acceptable treatment alternatives suggested by GPT. (**f**) Analysis of time spend (in seconds) by medical staff (human; check data availability and perform liver function analysis) and GPT versions (4, o1, o3, 5.4; liver function analysis, BCLC staging, treatment suggestion) with a short (SP) or long prompt (LP). Detailed statistical analyses are provided in the supplementary material.

For the Child–Pugh Score (Fig. 1a, supplemental Table 1), all GPT versions achieved accuracy levels above 85% for both short prompts (SP) and long prompts (LP), with a mean (± SD) of 93.2 ± 4.1%. For SP tasks, error rates decreased from 13.2% (version 4) to 5.7% (o1), 2.8% (o3), and 4.7% (5.4). A similar pattern was observed for LP tasks, with error rates of 14.2% (version 4), 6.6% (o1), 2.8% (o3), and 4.7% (5.4). However, , no significant differences were observed between SP and LP conditions (all p-values > 0.05).

Regarding error analysis, version 4 predominantly exhibited class 1 errors (source-/formula-based) during the LP task (71% of errors), whereas a broader distribution emerged in the SP task (class 1: 29%, class 2: 43%, class 4: 21%). As errors became less frequent in newer models (o1, o3, and 5.4), a shift in error pattern was observed, with the majority of remaining errors classified as class 2 (text/data interpretation). For o1 and o3, errors were almost exclusively class 2 across both SP and LP tasks. Similarly, version 5.4 showed predominantly class 2 errors in SP tasks (100%), while a small number of errors in LP tasks were distributed between class 1 (40%) and class 2 (60%).

For the ALBI grade (Fig. 1b, supplemental Table 2), all version–prompt constellations achieved accuracy levels above 85%, except for version 4 with the SP (40.6%), resulting in an overall mean (± SD) of 88.2 ± 18.7%. Statistical analysis (*p* < 0.001) revealed a significant difference among the eight version–prompt constellations. Specifically, version o1 exhibited significantly fewer errors than version 4 under both SP (2.8% vs. 59.4%) and LP tasks (0.9% vs. 12.3%). Similarly, version o3 demonstrated significantly fewer errors than version 4 for SP (0.0% vs. 59.4%) and LP tasks (0.9% vs. 12.3%). With version 5.4, high accuracy was likewise observed for SP tasks (3.8% error rate), comparable to other newer models. In contrast, version 5.4 showed a significantly higher error rate under LP conditions (14.2%) compared to version o1 (0.9%) and o3 (0.9%) and performed similar to the error rate of version 4 (12.3%).

When comparing prompt types, version 4 performed significantly better with LP tasks (12.3% error rate) than with SP tasks (59.4%). In contrast, no significant differences were observed between SP and LP tasks for the other versions. Notably, version o3 made significantly fewer errors with SP tasks (0.0%) than version 4 (12.3%) or 5.4 did with LP tasks (14.2%).

Regarding the error analysis, version 4 predominantly exhibited class 1 errors in the short prompt (SP) task (93%), whereas class 3 errors (calculation errors) were more common in the long prompt (LP) task (64%), followed by class 1 errors (36%). By contrast, the very few errors made by versions o1 and o3 were mainly class 1 errors. With version 5.4, a different pattern emerged: errors were predominantly classified as class 3, accounting for 87% of errors in LP tasks and 50% in SP tasks, with the remaining errors in SP tasks equally distributed between class 1 and class 3.

For the MELD score (Fig. 1c, supplemental Table 3), the overall accuracy showed a mean (± SD) of 74.2% ± 27.1%. Statistical analysis (*p* < 0.001) revealed a significant difference among the eight version–prompt constellations. Specifically, version 03 produced significantly fewer errors compared to version 4 (21.7) for the LP task (5.7%), and compared to version o1 (45.3%) and version 4 (88.7%) for the SP task (23.6%). With version 5.4, a marked improvement was observed for SP tasks, with an error rate of 3.8%, significantly lower than with all other models. In contrast, no statistically significant differences between models were observed for LP tasks, although a trend toward a higher error rate was noted for version 5.4 (14.2%), compared with o1 (3.8%) and o3 (5.7%), while remaining lower than version 4 (21.7%). When comparing prompt types, all earlier models (4, o1, o3) produced significantly fewer errors with LP tasks (21.7%, 3.8%, 5.7%) compared to SP tasks (88.7%, 45.3%, 23.6%). However, this pattern was not consistent for version 5.4 (LP: 14.2%, SP: 3.8%).

Regarding the error analysis for MELD scoring, earlier model versions predominantly exhibited class 1 errors in both the SP and LP tasks (ranging from 67 to 96%), followed by class 3 or 4 errors. In contrast, with version 5.4 a shift in the error pattern was observed. Here, errors were predominantly classified as class, accounting for 100% of errors in LP tasks and 75% in SP tasks, with the remaining 25% in SP tasks attributed to class 1 errors. Notably, no class 2 errors were observed for MELD scoring among any version in either task type.

### GPT for BCLC staging

Next, we challenged GPT to perform a BCLC staging based on the integrated reports containing clinical, laboratory and radiological data, which enabled to perform liver function analysis (see above) and assessment of tumor extension and clinical status of the patient.

In BCLC staging (Fig. 1d, supplemental Table 4), all version–prompt constellations achieved accuracy levels below 90%, with a mean (± SD) of 73.3 ± 13.8%. Statistical analysis (*p* < 0.001) showed a significant difference among the eight version–prompt constellations. Specifically, version o1 made significantly fewer errors than version 4 for both the SP task (27.4% vs. 53.8%) and the LP task (15.1% vs. 43.4%). Likewise, version o3 produced significantly fewer errors than version 4 in both the SP (14.2% vs. 53.8%) and LP (20.8% vs. 43.4%) tasks. Version 5.4 demonstrated significantly fewer errors compared to version 4 only under SP conditions (13.2% vs. 53.8%), while performing comparably to o3 (14.2%). When comparing prompt types, no consistent benefit of LP over SP was observed for BCLC staging. In fact, both o3 (20.8% vs. 14.2%) and 5.4 (25.5% vs. 13.2%) showed numerically higher error rates under LP compared with SP conditions; however, these differences did not reach statistical significance.

Regarding error analysis, class 2 errors (i.e., misclassification of radiological data) were the most frequent across all versions in both the short prompt (SP) and long prompt (LP) tasks, although a broad distribution across all error categories was observed. Only with version 5.4 under LP conditions, class 3 errors (i.e., errors in clinical data assessment, 56%) exceeded class 2 errors (41%).

Notably, the LP task increased class 1 errors in all versions (4: n = 11 [26%], o1: n = 3 [19%], o3: n = 7 [32%], 5.4: n = 1 [4%]) compared to the SP task (n = 6 [12%], n = 2 [7%], n = 4 [27%], n = 0 [0%], respectively). Further, the LP task reduced class 2 errors in version 4, o1 and o3 (LP: n = 13 [31%], n = 8 [50%], n = 7 [32%] vs. SP: n = 26 [50%], n = 12 [41%], n = 7 [47%], respectively), while it increased class 2 errors in version 5.4 (n = 11 [41%) vs. n = 9 [64%).

### GPT for therapeutic guidance

Next, we analyzed GPT’s treatment recommendations (Fig. 1e, supplemental Table 5) based on the integrated clinical, laboratory, and radiological reports, noting that each recommendation was contingent upon the model’s own BCLC stage determination. We then retrospectively compared these LLM-generated suggestions with decisions made by the interdisciplinary tumor board in the real-world setting. Statistical analysis (*p* < 0.001) revealed significant differences among the eight version–prompt constellations.

With version 4 using a SP, 48 of 106 patients (45.3%) received correct treatment suggestions, while 18.9% received acceptable alternatives and 35.8% received incorrect suggestions, which did not significantly differ from its LP scenario (51.9%, 17.9%, and 30.2%, respectively).

Version o3 with LP achieved the highest proportion of correct suggestions (84.0%) and was significantly higher than version 4 with SP (45.3%). Version 5.4 showed high correct suggestion rates under both SP (69.8%) and LP conditions (72.6%), without statistically significant differences compared to o3.

For false suggestions, o1 with LP (9.4%) and o3 with LP (10.4%) showed significantly lower error rates compared to version 4 with SP (35.8%) and LP (30.2%). Version 5.4 showed lower false suggestion rates than version 4 (SP: 17.0%; LP: 13.2%), although these differences were not consistently statistically significant.

When considering overall accuracy including acceptable alternatives, version o1 (90.6%) and o3 with LP (89.6%) with LP reached the highest agreement, followed by 5.4 with LP (86.8%) or SP (83.0%), and o3 with SP (85.8%). Version 4 showed the lowest agreement (SP: 64.2%, LP: 69.8%).

Interestingly, GPT suggested a ‘true’ or ‘acceptable alternative’ treatment in cases where the interdisciplinary tumor board retrospectively made an incorrect decision in 12.3% (SP) and 9.4% (LP) of cases with version 4, 12.3% and 14.2% with version o1,13.2% and 11.3% with version o3, and 13.2% and 10.4% for version 5.4, respectively. This discrepancy was likely due to incomplete data access or review by the tumor board, despite the relevant information being available in the electronic medical records. Interestingly, the human error rate is therefore close to the GPT error rate described above.

### Time efficiency and cost analysis

Time analysis (Fig. 1f, supplemental Table 6) revealed that trained medical staff required an average of 567.8 ± 59.5 s, which was significantly longer than all GPT version-prompt constellations, to verify the availability of mandatory clinical information and to gather relevant clinical, radiological, and laboratory data, including score calculations but not including BCLC staging or multidisciplinary tumor board discussions.

Among the different GPT model versions and prompt constellations, GPT-o3 with the SP yielded the fastest response time (53.2 ± 24.2 s). Version 4 required 64.0 ± 29.0 s with the SP and 61.4 ± 29.1 s with the LP. Meanwhile, GPT o1 required 105.8 ± 41.4 s susing the LP and 102.7 ± 45.5 s using the SP. The longest processing times were required by version 5.4 with 107.8 ± 30.1 s using the SP and 129.1 ± 47.3 s using the LP.

Cost analysis revealed that data interpretation by medical staff resulted in per-patient costs of $2.44 for a resident and $3.92 for a senior attending. In comparison, GPT incurred substantially lower costs, with version o3 and version 4 demonstrating the most economical performance (both SP / LP: $0.003 per case), followed by version o1 (SP/LP: $0.005) and version 5.4 (SP: $0.005; LP: $0,007). Depending on the used version or qualification of the physician, this corresponds to a cost reduction of approximately 300- to 1300-fold when using GPT instead of human interpretation.

## Discussion

In this study, we demonstrated that GPT can integrate clinical, radiological, and laboratory real-world data to calculate key liver function scores (Child–Pugh, MELD, and ALBI), assign BCLC stages, and provide treatment recommendations for HCC patients. These findings align with the recent surge of research indicating the potential of LLMs to assist in clinical workflows and research, including (radiological) report interpretation and overall decision-making support^9,24–29^. Importantly, the evaluated models differ in their underlying architecture and reasoning capabilities, with especially version o3 incorporating enhanced reasoning mechanisms. This may partly explain the observed performance results and should be considered when comparing results across studies. Here, GPT performance varied by model version and prompt length, highlighting that both model architecture and prompting strategy influence accuracy in clinically relevant tasks. While earlier models (version 4, o1, and o3) often showed improved performance with longer prompts for structured tasks such as liver function scoring, this effect was not consistent across all endpoints. Notably, for version 5.4, no systematic benefit of longer prompts was observed, and in some tasks performance varied depending on prompt configuration. These findings indicate that newer model versions do not uniformly yield improved performance and that the impact of prompt length is task- and model-dependent.

Short prompts, while convenient, commonly led to errors from insufficient instructions, hallucination or incomplete / incorrect formula integration especially occurring in older LLM versions, underscoring the importance of prompt engineering in AI-driven medical applications. These findings are congruent with other studies that emphasize the need for detailed input when using LLM to summarize and analyze complex medical data, with closed-source LLM however performing mostly better in unseen data tasks^9,30,31^. Notably, performance differed substantially between scores, with MELD calculation showing markedly lower accuracy compared to Child–Pugh and ALBI. This likely reflects the greater complexity of the MELD formula, which e.g. includes logarithmic transformations. Given the central role of MELD in organ allocation, these findings are clinically relevant and further emphasize that LLM-generated outputs must be carefully verified.

With respect to BCLC staging—a cornerstone in HCC management^2^—GPT showed generally lower accuracy than for liver function assessment likely reflecting the added complexity of integrating tumor burden, liver function, and performance status from heterogeneous clinical data (e.g., number and size of lesions, vascular invasion, ascites). Similar trends have been observed for other applications of clinical score computations by LLM^32^. Consistent with this, our error analysis revealed that misclassification of radiological findings was frequent, particularly under short-prompt conditions. While newer models demonstrated improved performance compared to earlier versions, differences among high-performing models (o1, o3, 5.4) were not consistently statistically significant.

For treatment recommendations, GPT frequently aligned with real-world multidisciplinary tumor board decisions but occasionally deviated, at times suggesting alternative strategies that were clinically acceptable—and in a small but relevant subset of cases, potentially more guideline-concordant. Notably, the error rate of LLM-generated recommendations was comparable to that observed in our real-world tumor board decisions, supporting thepotential role as a decision-support tool. The innovative potential of AI-driven advisement, particularly in revealing possible human oversights or misinterpretations of available or missing data, especially in settings increasingly challenged by economic constraints and workforce shortages^33–35^. However, the detected error rate also underscore that AI LLM tools, while promising, are not yet ready for autonomous clinical use^9,35,36^. Expert oversight remains essential to identify and correct potentially incomplete or inaccurate recommendations^37^.

Several limitations must be acknowledged. First, our retrospective design and single-center setting limit the generalizability of the findings. The present study should be interpreted as a single-center proof-of-concept study, and larger multicenter multilingual cohorts are required for confirmatory validation and for evaluating performance differences between model versions and prompting strategies. In this context, some degree of clinical ambiguity, e.g. regarding the defined gold standard by the expert panel, cannot be entirely excluded in such single center analysis. Second, while we excluded patients with incomplete datasets, real-world scenarios often involve missing information that can adversely affect both human and AI-based assessments. Here, LLM may have another important role to identify cases with missing data, e.g. prior to a multidisciplinary board avoiding a potential suboptimal decision due to incomplete data awareness of decision makers. In this context, a manual data collection from clinical systems was performed in our study accompanied by respective time consumption, showing the urgent need for improved data interfaces if LLM intend to effectively support clinical workflows in the future. Third, the rapid evolution of LLMs may render current versions obsolete; prospective validation with newer AI models is needed to confirm and extend these findings. Finally, we evaluated only German-language reports, and results may differ in other languages, and only one LLM was tested, while several other (biomedical) options are available. However, following the real-world design of this study, GPT is currently the most likely tool to be used by practitioners in health care and fine-tuned biomedical LLM may not perform better in such respective tasks^9,31,37^.

Despite these constraints, our study provides robust evidence that GPT can process heterogeneous patient data to perform HCC staging and treatment recommendations, especially when guided by comprehensive prompts, although currently it should only be considered as decision-support tool requiring human oversight rather than a standalone clinical solution. Additionally, our findings demonstrate a substantial time and cost advantage of GPT based clinical decision support over manual data processing by medical professionals. While human experts required an average of 9.3 min per patient solely for data availability checks and liver function analysis (excluding BCLC staging and board discussions), GPT models delivered complete responses in under 2 min across all versions and prompt formats with newer version (e.g. o3) significantly reducing the time need compared to older version. Cost analysis aligned with time savings, indicating that even the most expensive GPT setup was markedly more economical. Notably, our comparison did not account for the time or cost associated with data upload, prompt design or human validation of GPT outputs or LLM management within a clinical context, substantially limiting the generalizability of our analysis. However, our data highlights GPT’s potential efficiency in supporting routine oncological decision-making as a necessary scientific step towards clinically applicable AI-assisted workflows, though clinical oversight remains essential to ensure safety and accuracy.

Thus, LLM may tremendously assist towards the vision of integrative diagnostics to improve the value of patient care, where achieved outcome per resource spent is the critical factor^38,39^. Future research should explore integration of LLMs with electronic health records, continuous model fine-tuning for medical domains, and prospective trials evaluating clinical outcomes when AI-driven recommendations are incorporated into multidisciplinary care pathways^9^—all of which should take place under consideration of respective ethical standards^40^.

In conclusion, this study shows that GPT has the potential to assist data integration from clinical, laboratory and radiological data to assess liver function, perform BCLC staging and recommend guideline-based treatment in HCC patients.

## Supplementary Information

Below is the link to the electronic supplementary material.

Supplementary Material 1

## Data Availability

The datasets supporting the conclusions of this article are included within the article and its additional files.
