# Supplementary material for: Performance of GPT-based large language models in hepatocellular carcinoma stratification: liver function assessment, BCLC staging, and treatment recommendations
Source: Sci Rep. 2026 Jun 12;16:18342. doi: 10.1038/s41598-026-56992-7 (PMC13263330; doi:10.1038/s41598-026-56992-7)
Supplement: Supplementary file 1 — Supplementary Material 1 [file 41598_2026_56992_MOESM1_ESM.docx]

**Supplementary Material for**

**Performance of GPT-Based Large Language Models in Hepatocellular Carcinoma Stratification: Liver Function Assessment, BCLC Staging, and Treatment Recommendations**

Max Masthoff^1^, Amelie Zipser^1^, Michael Praktiknjo^2^, Jonel Trebicka^2^, Haluk Morgül^3^, Andreas Pascher^3^, Gesa Pöhler^1^, Michael Köhler^1^, Philipp Schindler^1^

1. Clinic for Radiology, University and University Hospital of Münster, Münster, Germany
2. Department of Internal Medicine B, University of Münster, Münster, Germany
3. Department of General, Visceral and Transplant Surgery, University of Münster, Münster, Germany

**Supplemental Table 1: Child-Pugh Score**

|  | | | | | | | |
| --- | --- | --- | --- | --- | --- | --- | --- |
|  | | Version | | | | | |
|  |  | SP_4 | | SP_o1 | | SP_o3 | |
|  |  | N | % | N | % | N | % |
| Correctness | false | 14_a_ | 13,2% | 6_a_ | 5,7% | 3_a_ | 2,8% |
|  | true | 92_a_ | 86,8% | 100_a_ | 94,3% | 103_a_ | 97,2% |
| Total | | 106 | 100,0% | 106 | 100,0% | 106 | 100,0% |

|  | | | | | | | |
| --- | --- | --- | --- | --- | --- | --- | --- |
|  | | Version | | | | | |
|  |  | SP_5.4 | | LP_4 | | LP_o1 | |
|  |  | N | % | N | % | N | % |
| Correctness | false | 5_a_ | 4,7% | 15_a_ | 14,2% | 7_a_ | 6,6% |
|  | true | 101_a_ | 95,3% | 91_a_ | 85,8% | 99_a_ | 93,4% |
| Total | | 106 | 100,0% | 106 | 100,0% | 106 | 100,0% |

|  | | | | | | | |
| --- | --- | --- | --- | --- | --- | --- | --- |
|  | | Version | | | | Total | |
|  |  | LP_o3 | | LP_5.4 | |  |  |
|  |  | N | % | N | % | N | % |
| Correctness | false | 3_a_ | 2,8% | 5_a_ | 4,7% | 58 | 6,8% |
|  | true | 103_a_ | 97,2% | 101_a_ | 95,3% | 790 | 93,2% |
| Total | | 106 | 100,0% | 106 | 100,0% | 848 | 100,0% |
| Each subscript letter denotes a subset of Version categories whose column proportions do not differ significantly from each other at the ,05 level. | | | | | | | |
|  |  |  |  |  |  |  |  |
|  | | | | | | | |

**Supplemental Table 2: ALBI Grade**

|  | | | | | | | |
| --- | --- | --- | --- | --- | --- | --- | --- |
|  | | Version | | | | | |
|  |  | SP_4 | | SP_o1 | | SP_o3 | |
|  |  | N | % | N | % | N | % |
| Correctness | false | 63_a_ | 59,4% | 3_b, c, d, e_ | 2,8% | 0_e_ | 0,0% |
|  | true | 43_a_ | 40,6% | 103_b, c, d, e_ | 97,2% | 106_e_ | 100,0% |
| Total | | 106 | 100,0% | 106 | 100,0% | 106 | 100,0% |

|  | | | | | | | |
| --- | --- | --- | --- | --- | --- | --- | --- |
|  | | Version | | | | | |
|  |  | SP_5.4 | | LP_4 | | LP_o1 | |
|  |  | N | % | N | % | N | % |
| Correctness | false | 4_b, c, d, e_ | 3,8% | 13_d_ | 12,3% | 1_c, e_ | 0,9% |
|  | true | 102_b, c, d, e_ | 96,2% | 93_d_ | 87,7% | 105_c, e_ | 99,1% |
| Total | | 106 | 100,0% | 106 | 100,0% | 106 | 100,0% |

|  | | | | | | | |  |
| --- | --- | --- | --- | --- | --- | --- | --- | --- |
|  | | Version | | | | Total | |  |
|  |  | LP_o3 | | LP_5.4 | |  |  |  |
|  |  | N | % | N | % | N | % |  |
| Correctness | false | 1_c, e_ | 0,9% | 15_b, d_ | 14,2% | 100 | 11,8% |  |
|  | true | 105_c, e_ | 99,1% | 91_b, d_ | 85,8% | 748 | 88,2% |  |
| Total | | 106 | 100,0% | 106 | 100,0% | 848 | 100,0% |  |
| Each subscript letter denotes a subset of Version categories whose column proportions do not differ significantly from each other at the ,05 level. | | | | | | | | |

|  |  |  |  |  |  |  |  | |
| --- | --- | --- | --- | --- | --- | --- | --- | --- |
|  | | | | | | | |  |

**Supplemental Table 3: MELD**

|  | | | | | | | |
| --- | --- | --- | --- | --- | --- | --- | --- |
|  | | Version | | | | | |
|  |  | SP_4 | | SP_o1 | | SP_o3 | |
|  |  | N | % | N | % | N | % |
| Correctness | false | 94_a_ | 88,7% | 48_b_ | 45,3% | 25_c_ | 23,6% |
|  | true | 12_a_ | 11,3% | 58_b_ | 54,7% | 81_c_ | 76,4% |
| Total | | 106 | 100,0% | 106 | 100,0% | 106 | 100,0% |

|  | | | | | | | |
| --- | --- | --- | --- | --- | --- | --- | --- |
|  | | Version | | | | | |
|  |  | SP_5.4 | | LP_4 | | LP_o1 | |
|  |  | N | % | N | % | N | % |
| Correctness | false | 4_d_ | 3,8% | 23_c_ | 21,7% | 4_d_ | 3,8% |
|  | true | 102_d_ | 96,2% | 83_c_ | 78,3% | 102_d_ | 96,2% |
| Total | | 106 | 100,0% | 106 | 100,0% | 106 | 100,0% |

|  | | | | | | | |
| --- | --- | --- | --- | --- | --- | --- | --- |
|  | | Version | | | | Total | |
|  |  | LP_o3 | | LP_5.4 | |  |  |
|  |  | N | % | N | % | N | % |
| Correctness | false | 6_d_ | 5,7% | 15_c, d_ | 14,2% | 219 | 25,8% |
|  | true | 100_d_ | 94,3% | 91_c, d_ | 85,8% | 629 | 74,2% |
| Total | | 106 | 100,0% | 106 | 100,0% | 848 | 100,0% |
| \| Each subscript letter denotes a subset of Version categories whose column proportions do not differ significantly from each other at the ,05 level. \| \| --- \| | | | | | | | |

|  |  |  |  |  |  |  |  |
| --- | --- | --- | --- | --- | --- | --- | --- |

**Supplemental Table 4: BCLC**

|  | | | | | | | |
| --- | --- | --- | --- | --- | --- | --- | --- |
|  | | Version | | | | | |
|  |  | SP_4 | | SP_o1 | | SP_o3 | |
|  |  | N | % | N | % | N | % |
| Correctness | false | 57_a_ | 53,8% | 29_b, c, d, e, f_ | 27,4% | 15_e, f_ | 14,2% |
|  | true | 49_a_ | 46,2% | 77_b, c, d, e, f_ | 72,6% | 91_e, f_ | 85,8% |
| Total | | 106 | 100,0% | 106 | 100,0% | 106 | 100,0% |

|  | | | | | | | |
| --- | --- | --- | --- | --- | --- | --- | --- |
|  | | Version | | | | | |
|  |  | SP_5.4 | | LP_4 | | LP_o1 | |
|  |  | N | % | N | % | N | % |
| Correctness | false | 14_d, f_ | 13,2% | 46_a, c_ | 43,4% | 16_b, d, e, f_ | 15,1% |
|  | true | 92_d, f_ | 86,8% | 60_a, c_ | 56,6% | 90_b, d, e, f_ | 84,9% |
| Total | | 106 | 100,0% | 106 | 100,0% | 106 | 100,0% |

|  | | | | | | | |
| --- | --- | --- | --- | --- | --- | --- | --- |
|  | | Version | | | | Total | |
|  |  | LP_o3 | | LP_5.4 | |  |  |
|  |  | N | % | N | % | N | % |
| Correctness | false | 22_b, d, e, f_ | 20,8% | 27_b, c, d, e, f_ | 25,5% | 226 | 26,7% |
|  | true | 84_b, d, e, f_ | 79,2% | 79_b, c, d, e, f_ | 74,5% | 622 | 73,3% |
| Total | | 106 | 100,0% | 106 | 100,0% | 848 | 100,0% |
| \| Each subscript letter denotes a subset of Version categories whose column proportions do not differ significantly from each other at the ,05 level. \| \| --- \| | | | | | | | |

|  |  |  |  |  |  |  |  |
| --- | --- | --- | --- | --- | --- | --- | --- |

**Supplemental Table 5: Treatment Suggestions**

|  | | | | | | | | |
| --- | --- | --- | --- | --- | --- | --- | --- | --- |
|  | | Version | | | | | |  |
|  |  | SP_4 | | SP_o1 | | SP_o3 | |  |
|  |  | N | % | N | % | N | % |  |
| Correctness | false | 38_a_ | 35,8% | 23_a, b, c_ | 21,7% | 15_b, c_ | 14,2% |  |
|  | alternative | 20_a_ | 18,9% | 19_a_ | 17,9% | 19_a_ | 17,9% |  |
|  | true | 48_a_ | 45,3% | 64_a, b_ | 60,4% | 72_b, c_ | 67,9% |  |
| Total | | 106 | 100,0% | 106 | 100,0% | 106 | 100,0% |  |

|  | | | | | | | |
| --- | --- | --- | --- | --- | --- | --- | --- |
|  | | Version | | | | | |
|  |  | SP_5.4 | | LP_4 | | LP_o1 | |
|  |  | N | % | N | % | N | % |
| Correctness | false | 18_a, b, c_ | 17,0% | 32_a, c_ | 30,2% | 10_b_ | 9,4% |
|  | alternative | 14_a_ | 13,2% | 19_a_ | 17,9% | 21_a_ | 19,8% |
|  | true | 74_b, c_ | 69,8% | 55_a, b_ | 51,9% | 75_b, c_ | 70,8% |
| Total | | 106 | 100,0% | 106 | 100,0% | 106 | 100,0% |

|  | | | | | | | |
| --- | --- | --- | --- | --- | --- | --- | --- |
|  | | Version | | | | Total | |
|  |  | LP_o3 | | LP_5.4 | |  |  |
|  |  | N | % | N | % | N | % |
| Correctness | false | 11_b_ | 10,4% | 14_b, c_ | 13,2% | 161 | 19,0% |
|  | alternative | 6_a_ | 5,7% | 15_a_ | 14,2% | 133 | 15,7% |
|  | true | 89_c_ | 84,0% | 77_b, c_ | 72,6% | 554 | 65,3% |
| Total | | 106 | 100,0% | 106 | 100,0% | 848 | 100,0% |
| Each subscript letter denotes a subset of Version categories whose column proportions do not differ significantly from each other at the ,05 level. | | | | | | | |

|  |  |  |  |  |  |  |  |
| --- | --- | --- | --- | --- | --- | --- | --- |

|  |
| --- |

**Supplemental Table 6: Time Analysis**

|  | **Human** | **4 – SP** | **o1 – SP** | **o3 - SP** | **5.4 -SP** | **4 – LP** | **o1 – LP** | **o3 - LP** | **5.4 -LP** |
| --- | --- | --- | --- | --- | --- | --- | --- | --- | --- |
| **Mean (in s)** | 567.8 | 64.0 | 102.7 | 53.2 | 107.8 | 61.4 | 105.8 | 62.8 | 129.1 |
| **Std. Deviation** | 59.5 | 29.0 | 45.5 | 24.2 | 30.1 | 29.1 | 41.4 | 22.5 | 47.3 |
|  | **ANOVA** p-value: < 0.0001  **Post-hoc Tukey Test** | | | | | | | |  |
| **Human** | n/a | p < 0.05 | p < 0.05 | p < 0.05 | p < 0.05 | p < 0.05 | p < 0.05 | p < 0.05 | p < 0.05 |
| **4 – SP** |  | n/a | p < 0.05 | ns | p < 0.05 | ns | p < 0.05 | ns | p < 0.05 |
| **o1 – SP** |  |  | n/a | p < 0.05 | ns | p < 0.05 | ns | p < 0.05 | p < 0.05 |
| **o3 - SP** |  |  |  | n/a | p < 0.05 | ns | p < 0.05 | ns | p < 0.05 |
| **5.4 - SP** |  |  |  |  | n/a | p < 0.05 | ns | p < 0.05 | p < 0.05 |
| **4 – LP** |  |  |  |  |  | n/a | p < 0.05 | ns | p < 0.05 |
| **o1 – LP** |  |  |  |  |  |  | n/a | p < 0.05 | p < 0.05 |
| **o3 - LP** |  |  |  |  |  |  |  | n/a | p < 0.05 |
| **5.4 - LP** |  |  |  |  |  |  |  |  | n/a |

SP: short prompt; LP: long prompt; n/a: not applicable; ns: not significant (p-value > 0.05).
